# Supplementary material for: Differential Dependency of Human Pancreatic Cancer Cells on Targeting PTEN via PLK 1 Expression
Source: Cancers (Basel). 2020 Jan 23;12(2):277. doi: 10.3390/cancers12020277 (PMC7072440; doi:10.3390/cancers12020277)
Supplement: Supplementary file 1 [file cancers-12-00277-s001.pdf]

Supplementary Materials: Differential Dependency of Human Pancreatic Cancer Cells on Targeting PTEN via PLK 1 Expression

Jungwhoi Lee, Jungsul Lee, Woogwang Sim and Jae-Hoon Kim

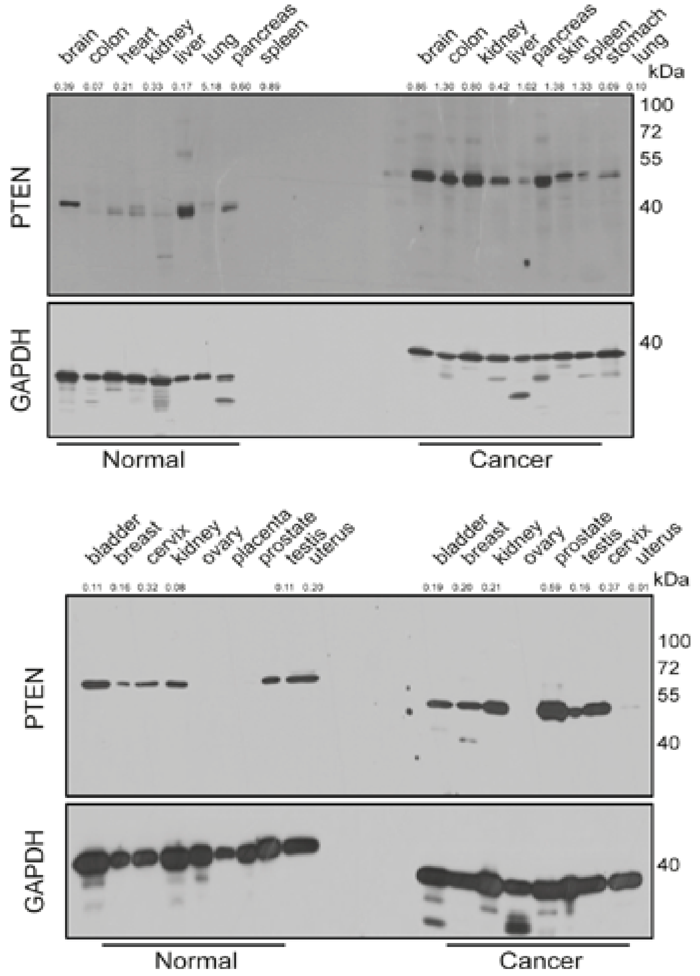

Figure S1. Expressions of phosphatase and tensin homolog (PTEN) in human pancreatic cancer. Human Normal Tissue Blot I and Human Tumor Tissue Blot I have been used to determine the expression of PTEN in various normal and tumour tissues. GAPDH has been used as a control. Data represent two individual experiments.

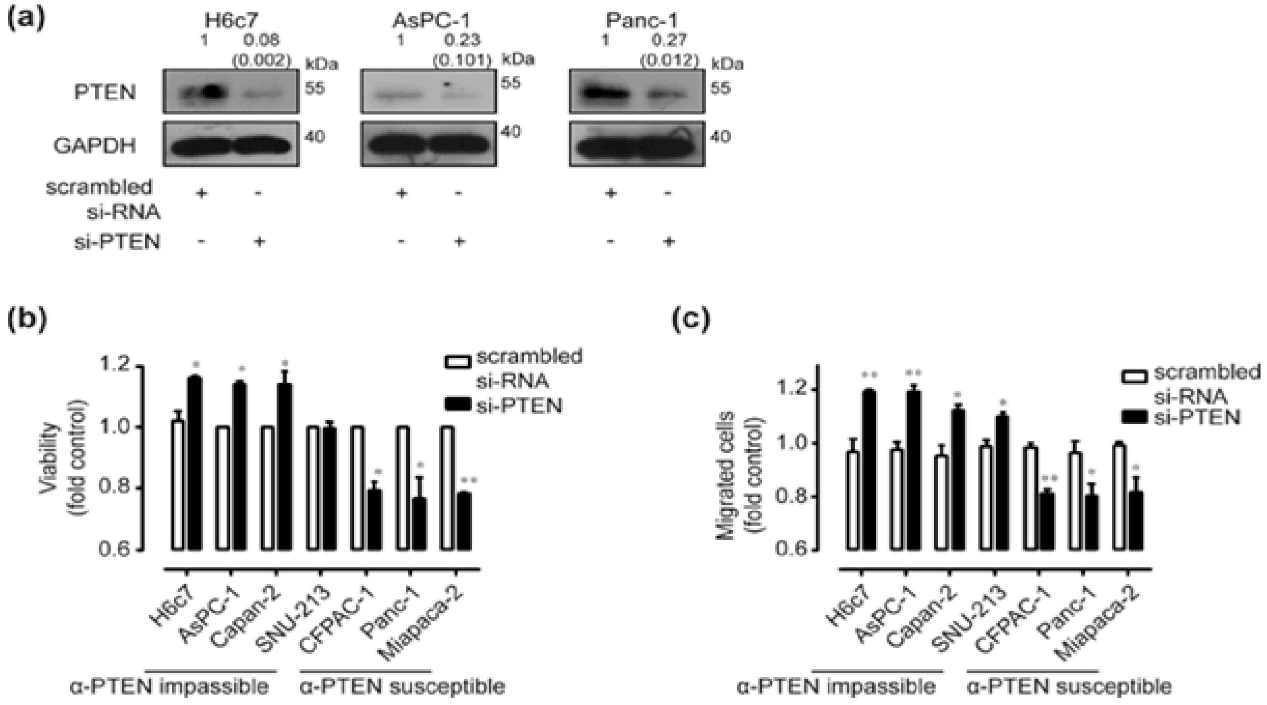

Figure S2. *In vitro* effects of phosphatase and tensin homolog (PTEN) blockade in human pancreatic cancer cells. (a) H6c7, AsPC-1 and Panc-1 cells were transfected with scrambled or PTEN-specific siRNA for 72 h. PTEN and GAPDH protein levels were analysed by Western blot. Data is representative of three individual experiments. (b) H6c7, AsPC-1, Capan-2, Miapaca-2, SNU-213, CFPAC-1, Panc-1 and Miapaca-2 cells were transfected with scrambled or PTEN-specific siRNA for 72 h. The viability was measured by WST-1 assay (*P*-value evaluated with Student's *t* test, and data represent three individual experiments, $^{*}p < 0.05$, $^{**}p < 0.01$). (c) H6c7, AsPC-1, Capan-2, Miapaca-2, SNU-213, CFPAC-1, Panc-1 and Miapaca-2 cells were transfected with scrambled or PTEN-specific siRNA. After 48 h of transfection, the cells were exposed to serum-starved conditions. After 24 h of serum starvation, migrated cells were evaluated using the Transwell-migration assay for 6 h (*P*-value evaluated with Student's *t* test, and data represent three individual experiments, $^{*}p < 0.05$, $^{**}p < 0.01$).

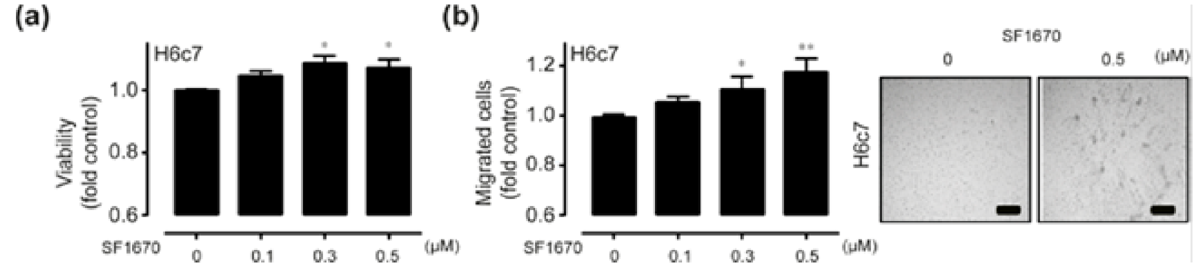

Figure S3. *In vitro* effects of phosphatase and tensin homolog (PTEN) blockade in human normal pancreatic duct epithelial H6c7 cells. (a) H6c7 cells were incubated with varying doses of SF1670 for 72 h. The viability was measured by WST-1 assay ($n = 3$; Tukey's *post-hoc* test was used to detect significant differences in ANOVA, $p < 0.0001$; asterisks indicate a significant difference compared with 0% inhibition, Data is representative of three individual experiments, $^{*}p < 0.05$). (b) Left, H6C7 cells were incubated with varying doses of SF1670 for 6 h. The migration activities were evaluated using the transwell-migration assay ($n = 3$; Tukey's *post-hoc* test was used to detect significant differences in ANOVA, $p < 0.0001$; asterisks indicate a significant difference compared with 0% inhibition, Data is representative of three individual experiments, $^{*}p < 0.05$, $^{**}p < 0.01$). Right, Representative image of trans-well migration assay (scale bar = 50 μm).

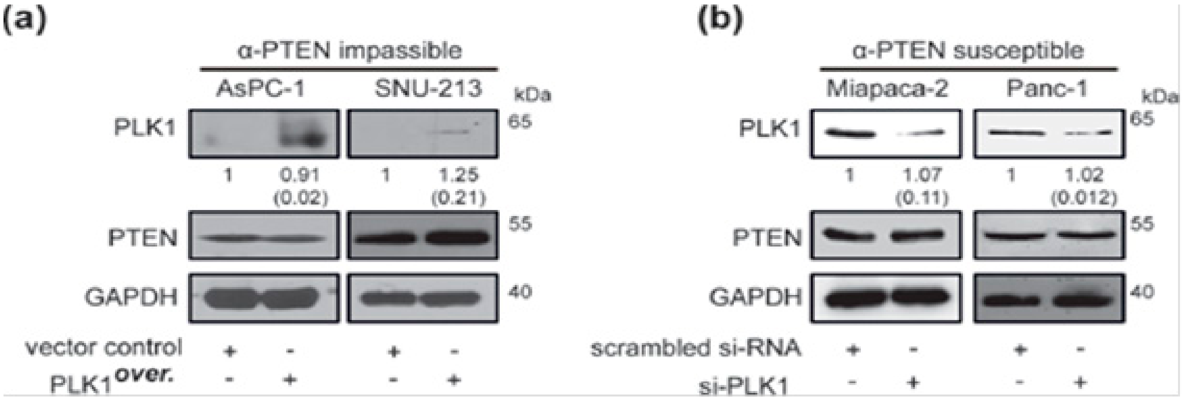

Figure S4. Expression of polo-like kinase 1 (PLK1) as a companion biomarker with phosphatase and tensin homolog (PTEN) expression in pancreatic cancer. (**a**) After 48 h of transfection with the vector control or PLK1 overexpression construct, AsPC-1 and SNU-213 cell lysates were subjected to Western blot analysis using antibodies specific for PLK1 and PTEN. GAPDH was used as a control. (**b**) Miapaca-2 and Panc-1 cells were transfected with scrambled or PLK1-specific siRNA. After 48 h of transfection, Miapaca-2 and Panc-1 cell lysates were subjected to immunoblot analysis using antibodies specific for PLK1 and PTEN. GAPDH was used as a control.

#	Specimen ID	Age	Sex	Ethnicity	Height (cm)	Weight (kg)	Stage
C1	417709	60	Male	N/A	N/A	N/A	Stage 1
C2	447604	60	Male	N/A	N/A	N/A	Stage 2
C3	282454A3	48	Male	Asian	168	51	T3NXM0
C4	265359A2	60	Male	Asian	167	57	T4NXM0
C5	339106A3	61	Female	Asian	160	59	T3N0M0
C6	277682	65	Male	African American	185	90	T3N0MX
C7	305533A2	47	Female	Asian	140	38	T4N0M1
C8	T1210496	54	Male	Caucasian	N/A	N/A	T3N1MX
C9	291891A3	60	Male	Asian	167	49	T3NXM1
C10	339106A2	61	Female	Asian	160	59	T3N0M0
C11	437486	74	Male	Caucasian	N/A	N/A	Stage 4
C12	265359A3	60	Male	Asian	167	57	T4NXM0

Figure S5. Clinical information of whole blood samples from twelve pancreatic cancer patients.

Figure 1

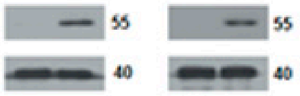

Figure 2

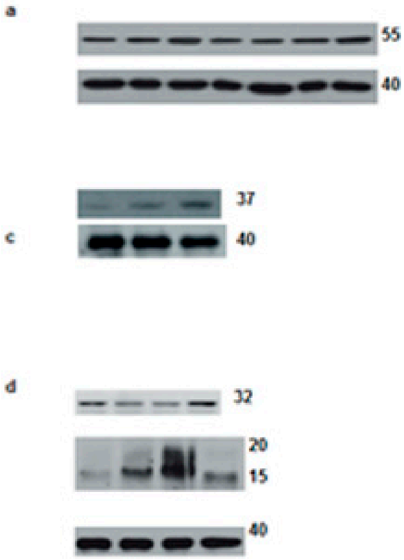

Figure 3

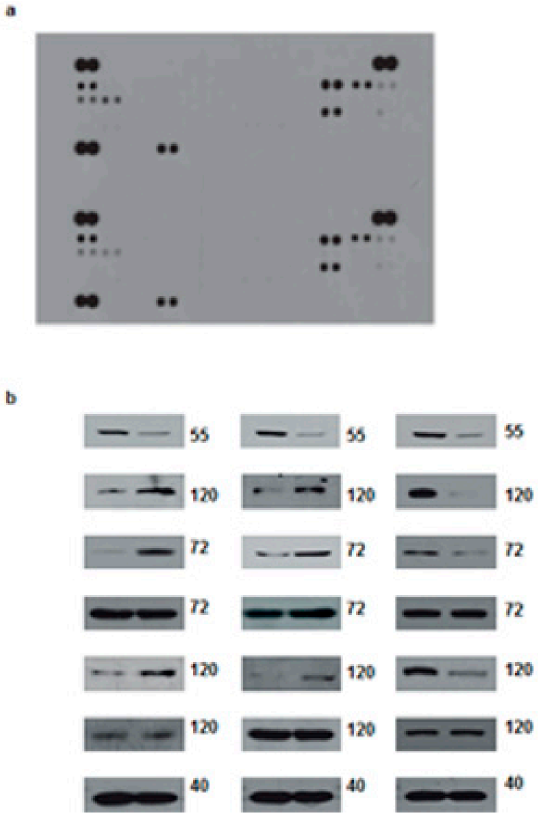

Figure 4

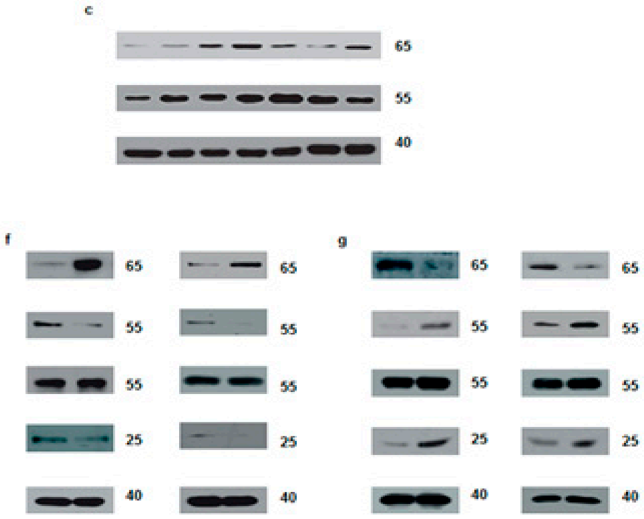

Figure 5

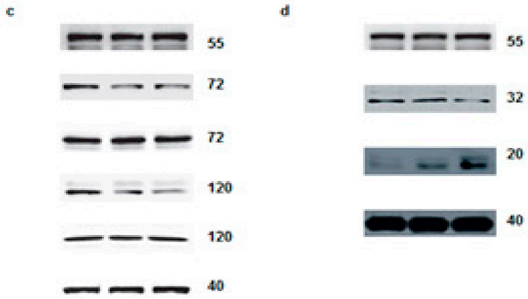

Figure 7

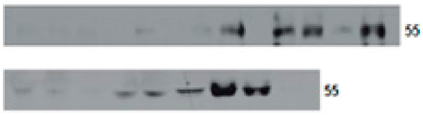

Figure S1

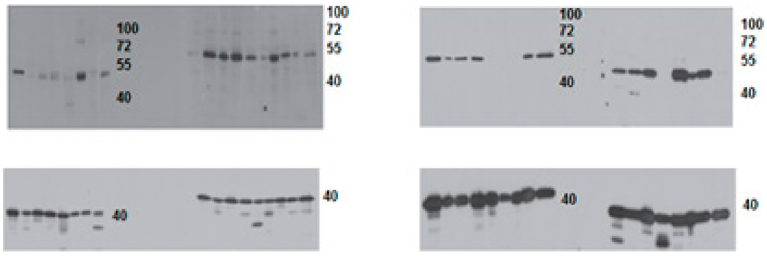

Figure S2

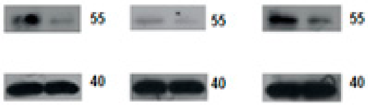

Figure S6. Original blot figures.

© 2020 by the authors. Licensee MDPI, Basel, Switzerland. This article is an open access article distributed under the terms and conditions of the Creative Commons Attribution (CC BY) license (http://creativecommons.org/licenses/by/4.0/).
